# Mammography datasets and artificial intelligence for breast cancer detection: challenges, bias, and future directions

**DOI:** 10.3389/frai.2026.1933770

**Published:** 2026-10-20

**Authors:** A. Kaja Mohideen, P. N. Senthil Prakash, M. K. Priyadharshini

**Affiliations:** School of Computer Science and Engineering (SCOPE), Vellore Institute of Technology, Chennai, India

**Keywords:** breast cancer detection, computer-aided diagnosis, deep learning, machine learning, mammography, transfer learning, vision transformers

## Abstract

**Background:**

Breast cancer is still one of the main causes of death in women from cancer worldwide, and early detection is crucial. Mammography is the primary imaging modality for breast cancer screening and recent advances in artificial intelligence (AI) have significantly enhanced automated breast cancer detection. This review takes a data-centered approach, examining key characteristics of public datasets, the contributions of mammography data to AI for breast cancer detection, and the implications for clinical generalizability. This review, however, takes a data-centered approach, critically analyzing characteristics of publicly available mammography datasets, and the role of mammography data to the development, performance and clinical generalization of AI models for the detection of breast cancer. The datasets are compared on the following aspects: dataset size, quality of the annotations, imaging modality, clinical metadata, dataset accessibility and demographic diversity. Analysis reveals that the quality, diversity and representativeness of the data used are critical for the performance and generalizability of AI models, ranging from the traditional machine learning era to the current era of deep learning, transformer-based systems, multimodal learning, explainable AI and privacy-preserving learning.

**Conclusion:**

The results show that, apart from the development of AI model architectures, it is a necessity to have standardized, diverse and clinically representative datasets to make further progress in AI-assisted mammography. The review emphasizes the importance of establishing a comprehensive benchmark dataset for mammography that is large and diverse to enhance reproducibility, to fairly evaluate models, and to develop strong, clinically viable AI systems for breast cancer detection.

## 1 Introduction

Breast cancer is a life-threatening condition, and is one of the most common causes of cancer-related death in women around the world. The sooner the diagnosis is made, the easier the cure. Clinically proven data show that the prognosis of cancer is good at an early stage. So, there is a critical need for scientifically efficient and effective screening methods. Mammography has been the most successful and widespread imaging technique for breast cancer screening. Screening can identify abnormalities like masses, calcifications, and architectural distortions unique to breast cancer. Despite being the standard and reliable method for breast cancer screening, mammography is associated with challenges in analyzing mammograms, including the density of the breast, the quality of the image, the shape of the lesion, and the experience of the radiologist. These challenges often lead to ambiguity in diagnosis, resulting in false-positive and false-negative results, extra imaging procedures, and unnecessary biopsies when clinically indicated. With the advent of automated and AI-assisted systems, an opportunity has emerged to support radiologists in mammographic image analysis, ensuring consistency and accelerating the process.

The problem of automated detection of breast cancer from mammography is addressed in the literature by developing computer-aided diagnosis systems based on the principles of machine learning and deep learning. In its initial stages, the research focused on the development of conventional artificial intelligence algorithms for automatic detection of tumors through manual description of significant image features. Despite being demonstrated as effective in controlled settings, these methods have limited applicability in practice due to difficulties in processing complex high-resolution mammographic images (Xie et al., 2016).

The emergence of deep learning has opened new opportunities for researchers through the use of convolutional neural networks. These networks allow for the automatic identification of significant features from raw data. This technology enables a more accurate medical diagnosis of diseases, including breast cancer: classification, detection, and segmentation of breast lesions on mammographic images (Kavitha et al., 2022; Oza et al., 2023; Hassan et al., 2024; Karaca Aydemir et al., 2025).

At the same time, the main direction of scientific research is the improvement of existing technologies by introducing hybrid models and exploring the effectiveness of transformer-based architectures and ensemble learning techniques for medical imaging tasks. The development of new learning methods, including multimodal learning, is also of interest to researchers as a way to improve the accuracy of breast cancer detection algorithms. Federated learning is a rapidly growing research area that is considered a promising direction for protecting patient privacy, with its potential applications in medical data processing (Atrey et al., 2023, 2024).

Despite the wide range of promising methods, it should be noted that the development of artificial intelligence systems for breast cancer detection is largely dependent on the characteristics of the available data sets of mammographic images. Most of the literature reviews use publicly available data sets. The main publicly available data sets include MIAS, DDSM/CBIS-DDSM, and INbreast, which are often used by researchers in the field. However, their use is complicated by significant differences in the characteristics of the data sets, which are not always publicly available. Moreover, most studies do not report the statistical significance of the results when applied to different data sets. Variations in data set characteristics, such as sample size, age, ethnicity, and imaging modalities, affect the validity of generalizing study results and the ability to build robust and reliable AI computer-aided diagnosis systems (Murtaza et al., 2020; Jeba Prasanna Idas et al., 2025; Hassan et al., 2022).

The literature is abundant with research on breast cancer detection through digital mammography but there remain areas where research is still lacking. The inconsistency of annotations in publicly available data sets is one of the challenges which makes learning with artificial intelligence (AI) models and analyzing the outcome of such models more difficult. Although there is a wealth of research on breast cancer detection from mammography, there is a need for annotated data sets containing a greater number of diverse images from different populations to ensure greater representation in terms of race/ethnicity and age. Furthermore, it remains challenging to find a consistent approach to perform the validation of computer-aided diagnosis systems both within and between different data sets and in external validation studies.

This paper differs from previous reviews that concentrate on the algorithmic techniques of breast cancer detection via digital mammography, and examines the attributes of public available breast data sets and the effect they have on the performance of an AI system. The study discusses the challenges associated with publicly available data sets used in scientific research on breast cancer screening and puts forward recommendations for solving them. This study states the need to collect a broad benchmark of annotated mammographic images, which will serve as a basis for subsequent research into automated breast cancer detection technology. Finally, this review outlines the promising research directions, including the use of multimodal data sets and methods of federated learning for medical imaging.

The main contributions of this review are as follows:

(a) Analysis of publicly available data sets used for breast cancer detection from mammography.
(b) Critical assessment of the current state of research on artificial intelligence technology for automating and accelerating the process of mammography.
(c) Identification of current trends and research needs, as well as obstacles to standardized benchmarking.
(d) The need for annotated benchmark mammography data sets for building clinical decision-making support systems.

Research direction recommendations, including those related to multimodal data sets, external validation, and standardized benchmarking.

The rest of the paper is organized as follows. Section 2 describes the review methodology. Sections 3 and 4 discuss datasets and annotation strategies for mammography, respectively. Section 5 reviews critically AI models for breast cancer detection. Open challenges, future research priorities, and emerging research trends are discussed in Sections 6 and 7. Finally, Section 8 emphasizes the need for a globally diverse benchmark dataset of mammograms and concludes the review.

## 2 Methodology

In this paper, a structured narrative review of the recent advances in the mammogram datasets and the detection of breast cancer using machine learning (ML) and deep learning (DL) techniques is presented. The goal is to collate and review the literature to extract trends in the development of datasets, model evolutions, performance assessment, and challenges to clinical deployment. Special attention is given to the factors that impact the ability of artificial intelligence (AI) models to be robust and generalizable, such as the size of the dataset, the quality of the annotations, the diversity of the demographics, and geographic origin.

### 2.1 Literature search strategy

The literature search strategy used in looking for the eligible articles is elaborated, with databases searched and keywords used. Further, details of articles included in the review and excluded are provided along with information about the time frame of the search. Relevant literature was identified by searching for articles published from 2016 to 2025 in scientific databases, such as IEEE Xplore, SpringerLink, ScienceDirect, PubMed, and Google Scholar. The search terms used were: *breast cancer detection* and *mammography* combined with *breast cancer detection machine learning, breast cancer detection deep learning, breast cancer detection transfer learning, breast cancer detection Vision Transformer, breast cancer detection computer-aided diagnosis*, and *mammography datasets*. Articles from the selected period were reviewed and further articles were found in the bibliography of the selected articles to ensure that an article covered the topic as comprehensively as possible.

### 2.2 Study selection

The selection process used in determining eligibility of the articles for inclusion in the review was provided and a description of the screening stages was provided. The criteria for the selection of studies for the review were: the study focused on detection of breast cancer using mammography and the study was relevant to the research questions. Preference was placed on the articles that discuss the machine learning and deep learning algorithms used to detect, classify or computer-aided diagnosis (CAD) breast cancer from mammographic images. Special emphasis was given to articles that describe computer-aided diagnosis methods that have been applied to the publically available mammography data sets. Articles that focus exclusively on imaging modalities other than digital mammography (e.g., ultrasound, magnetic resonance imaging, histopathology, and thermography) were excluded from the review, as were articles with insufficient methodological descriptions or quantitative evaluations. See Figure 1 for details.

**Figure 1:**
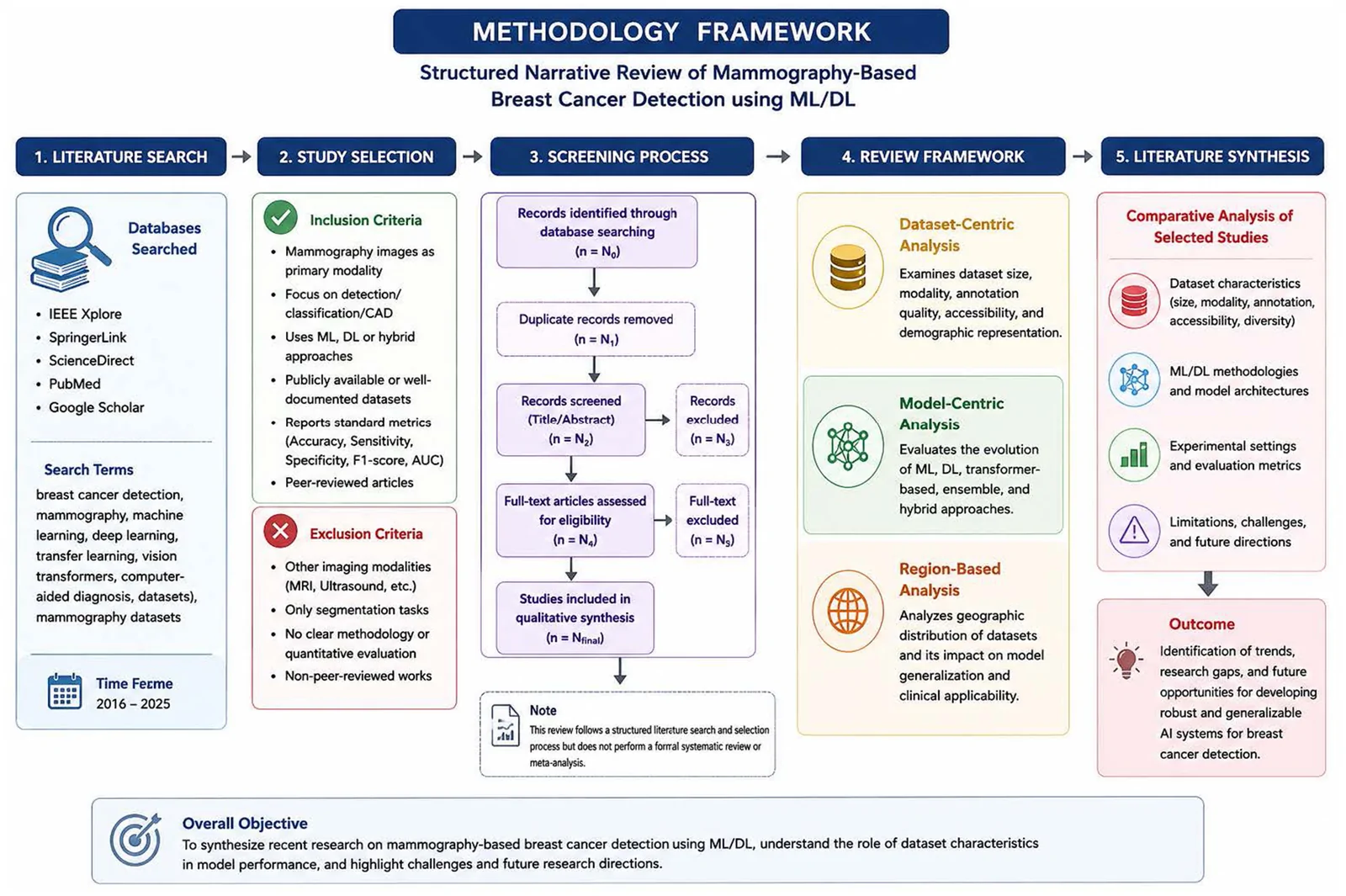
Framework of the structured narrative review adopted for the analysis of mammography-based breast cancer detection studies.

### 2.3 Literature synthesis

A description is given of the process of organizing and synthesizing the data extracted from the selected articles, including the methods used to analyze and classify the information. Each of the included studies was examined in terms of the four aspects listed below, and the information was synthesized to identify trends, research needs, and directions for future research:

(i) characteristics of the mammography data sets used in the study: size, type of imaging modality, annotation, class distribution, availability, and demographic information;
(ii) machine learning and deep learning methods used to analyze mammographic images;
(iii) experimental design and evaluation metrics;
(iv) limitations and research needs identified by the authors.

Special attention was paid to information on the characteristics of the data sets used, which directly impact the performance of the methods under study. The studies were grouped according to the research questions and the technical aspects of solving the problems posed. Particular attention was paid to trends in the use of different machine learning methods, including traditional approaches, convolutional neural networks, transfer learning, vision transformers, multimodal learning, explainable artificial intelligence, and privacy-preserving methods such as federated learning. The findings of the literature synthesis are presented in the sections devoted to the discussion of research trends and research needs.

### 2.4 Assessment of dataset suitability for deep learning architectures

The suitability of each dataset for CNN- and Transformer-based models was qualitatively assessed using four dataset characteristics: dataset size, image modality, annotation availability, and label richness. CNN suitability was primarily determined by the availability of sufficient images and annotations for supervised learning, whereas Transformer suitability additionally considered dataset scale and annotation richness, as these models generally benefit from larger and more diverse training data.

Based on these characteristics, each dataset was assigned a qualitative rating of *Low, Medium, High*, or *Very High*. *Low* indicates limited data or annotations; *Medium* indicates suitability with the possible need for transfer learning or augmentation; *High* indicates favorable conditions for supervised deep-learning development; and *Very High* indicates large-scale datasets with rich annotations and clinical information.

## 3 Overview of mammography datasets

Availability of high-quality datasets is a key factor in the development of the reliable computer-aided diagnosis (CAD) systems to detect breast cancer. Datasets serve as the foundation to train and test the machine learning and deep learning models in the context of mammography. Nevertheless, although the AI in healthcare has made a massive progress, the current mammography datasets have multiple limitations in size, diversity, and the quality of annotations.

This section gives an overview of popular publicly available datasets in a comprehensive manner, after which their limitations are critically analyzed.

### 3.1 Widely used public mammography datasets

Public mammography datasets form the foundation for the development and benchmarking of AI-based breast cancer detection systems. Over the past three decades, these datasets have evolved from small film-based collections with image-level labels to large-scale digital repositories containing lesion annotations, BI-RADS assessments, and clinical metadata. While early datasets primarily supported traditional machine learning methods, recent repositories facilitate the development of deep learning, transformer-based, and multimodal diagnostic models. Table 1 summarizes the characteristics of the major publicly available mammography datasets. Figure 2 shows the trends in the use of mammography datasets for AI-based breast cancer detection research used in this study.

**Table 1:** Comparison of major public mammography datasets.

Dataset	Year	Country	Patients	Images	Modality	Annotation	Public	Primary applications
MIAS (Suckling et al., 2015)	1994	UK	161	322	Film	Image-level labels	Yes	Classification
DDSM (Heath et al., 2000)	1997	USA	2,620	10,480	Film	ROI + Pathology	Yes	Classification, Detection
CBIS-DDSM (Lee et al., 2017)	2017	USA	1,566	3,103	Digitized Film	ROI Segmentation + Pathology	Yes	Classification, Detection, and Segmentation
INbreast (Moreira et al., 2012)	2012	Portugal	115	410	FFDM	Pixel-level Masks	Yes	Detection, Segmentation
BCDR (Lopez et al., 2012)	2012	Portugal	1,734	7,315	Film/Digital	Clinical Labels	Yes	Classification
VinDr-Mammo (Nguyen et al., 2023)	2023	Vietnam	5,000 Exams	20,000 Images	FFDM	BI-RADS + Bounding Boxes	Yes	Classification, Detection
CMMD (Cai et al., 2023)	2023	China	1,775	3,712	FFDM	Pathology Labels	Yes	Classification
CSAW-M (Sorkhei et al., 2021)	2021	Sweden	10,020	10,020	FFDM	Masking Annotation	Restricted	Risk Prediction
OMI-DB (Halling-Brown et al., 2020)	2020	UK	17,282	Large-scale	FFDM	ROI + Clinical Metadata	Restricted	Longitudinal Analysis
DMID (Oza et al., 2024a)	2023	India	–	510	FFDM	Lesion + Clinical Metadata	Yes	Detection, Segmentation, and Classification

**Figure 2:**
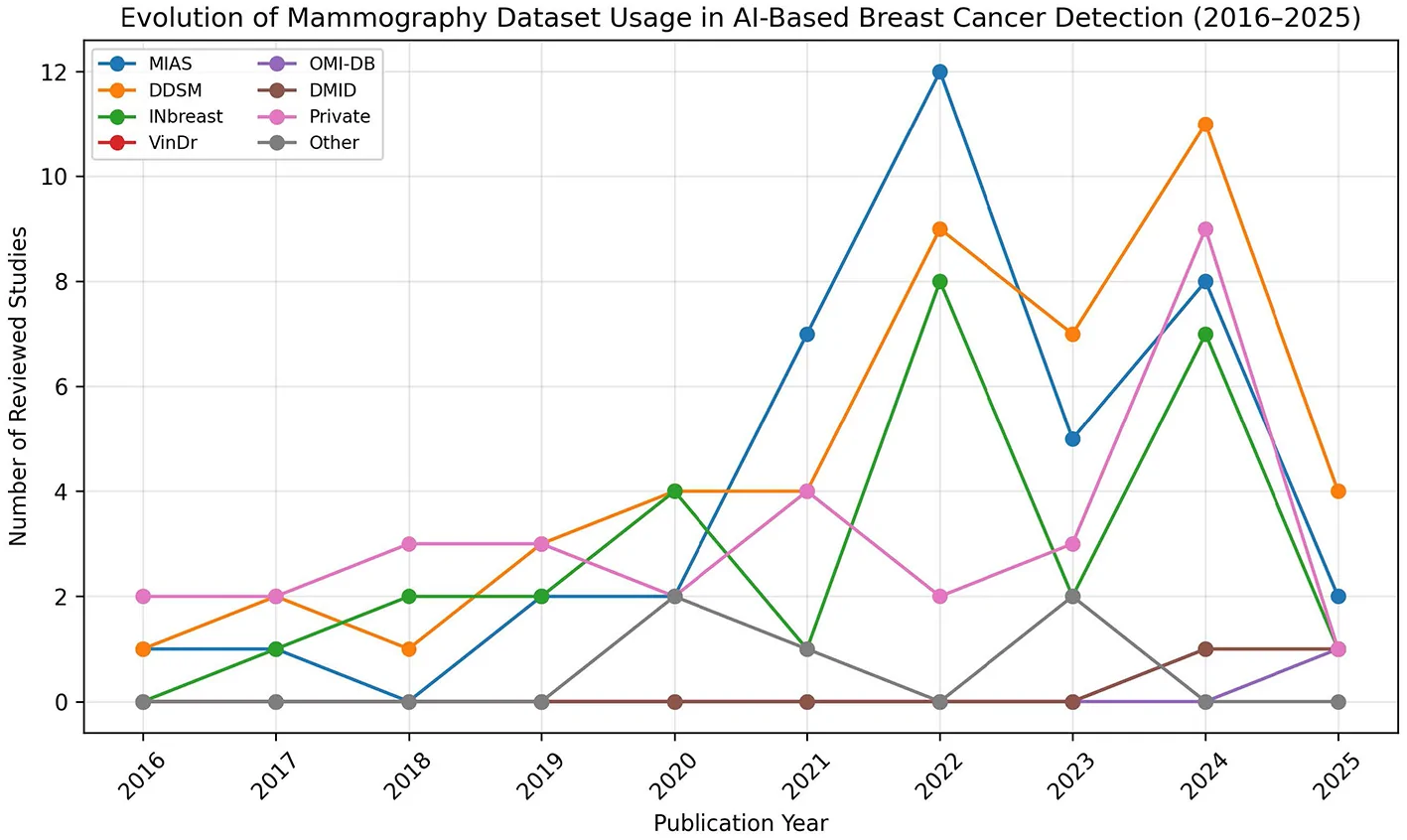
Trends in the use of mammography datasets for AI-based breast cancer detection research from 2016 to 2025.

#### 3.1.1 MIAS dataset

The MIAS dataset (Suckling et al., 2015) is one of the publicly available mammography datasets, which contains 322 mammograms categorized as normal, benign, and malignant. Due to its simplicity and general availability, MIAS has been widely used as a benchmark for conventional machine learning algorithms and lightweight CNN models (Melekoodappattu et al., 2023; Aymaz, 2024; Ramesh et al., 2022; Patil et al., 2022; Al-Fahaidy et al., 2022; Alruwaili and Gouda, 2022; Samee et al., 2022; Kanya Kumari and Naga Jagadesh, 2024; Kalam and Thomas, 2024; Ghafariasl et al., 2025).

However, MIAS also has some significant limitations in terms of modern AI applications. Compared with other datasets, MIAS has a relatively small number of samples, limited demographic variation, and does not provide detailed annotations for lesions. As a result, most of the recent studies adopted extensive data augmentation or combined MIAS with other large-scale datasets for training.

#### 3.1.2 DDSM and CBIS-DDSM datasets

Digital Database for Screening Mammography (DDSM) (Heath et al., 2000) and its curated version, CBIS-DDSM, (Lee et al., 2017) are two of the most widely used benchmark datasets for mammography analysis, which provide a significantly larger number of images, standard lesion annotations, pathological labels, and training protocols compared with MIAS.

A variety of AI applications, including image classification, lesion detection, and computer-aided diagnosis supported by CNNs, object detectors, and vision transformers, have been developed based on these datasets (Al-Masni et al., 2018; Shen et al., 2019; Montaha et al., 2021; Azour and Boukerche, 2022; Chugh et al., 2024). However, both datasets were created mostly from digitized film mammograms provided by western populations, which may lead to image quality variation and demographic bias. These limitations are not conducive to the application of modern deep learning methods that require extensive data with demographic variety.

#### 3.1.3 INbreast dataset

INbreast dataset (Moreira et al., 2012) is a high-quality full-field digital mammography (FFDM) dataset that provides pixel-level annotations of lesions and pathological findings. This dataset is widely used for lesion detection, segmentation, and explainable computer-aided diagnosis systems. Several studies have shown that INbreast can be used for object detection and integrated diagnostic frameworks (Ribli et al., 2018; Al-Antari et al., 2018).

However, it contains a relatively small number of cases and represents a limited patient population, making it suitable for model validation and fine-tuning rather than training large-scale deep learning models from scratch.

#### 3.1.4 Recent large-scale public datasets

To address the limitations of earlier benchmark datasets, several large-scale repositories have recently been introduced. OMI-DB (Halling-Brown et al., 2020) provides longitudinal multi-center screening data from the United Kingdom with detailed lesion annotations, making it suitable for temporal measurements and risk prediction. VinDr-Mammo (Nguyen et al., 2023) contains 5,000 full-field digital mammography examinations with breast-level and lesion-level annotations, supporting multiple downstream tasks including detection, classification, and localization. The Chinese Mammography Database (CMMD) (Cai et al., 2023) consists of histopathological labels and molecular subtype information, enabling precision medicine research, although it lacks region-of-interest annotations. Similarly, the CSAW and CSAW-M datasets (Dembrower et al., 2020; Sorkhei et al., 2021) provide longitudinal screening data and masking annotations that enable studies on breast density and interval cancer detection.

Another important and recent dataset is the Digital Mammography Imaging Dataset (DMID) (Oza et al., 2024a,b), originating from the Indian population. In addition to lesion-level annotations, DMID includes clinical metadata such as patient age, imaging views, and diagnostic labels, making it particularly suitable for developing region-specific AI systems by improving model generalization across diverse populations.

#### 3.1.5 Private and institutional datasets

Most of the recent studies from the reviewed literature use private or institutional datasets to overcome the limitations of existing benchmarks. Large clinical datasets generally provide patient diversity, higher-quality annotations, and clinical metadata compared to public datasets, enabling the development of more robust diagnostic models (Samala et al., 2018; Mohamed et al., 2018; Hassan et al., 2020; Nica et al., 2021; Pesapane et al., 2023). But lack of public access can make it hard to reproduce, validate, and provide a standard benchmark for comparing studies.

Mammography data sets have clearly followed a trend of increasingly large, more well-annotated, and richer data sets for clinical purposes. However, significant obstacles still remain in the way of the development of clinically generalizable AI models, such as demographic bias, the lack of multi-center representation, inconsistencies in annotation standards, and access to institutional datasets.

### 3.2 Comparative analysis of mammography datasets

The comparative evaluation shows several interesting trends in the development of mammography databases. First, the size of the datasets has grown significantly over the last 30 years, starting with a few hundred mammograms in MIAS and moving to tens of thousands of data sets in the new repositories like VinDr-Mammo, OMI-DB, and DMID. The rise has enabled the use of ever-more complex AI models, such as deep convolutional networks and Vision Transformers that need large and diverse training data sets.

Second, they have created a much better version of the annotation. The original datasets mostly included image-level diagnosis labels, while current repositories offer lesion segmentation masks, bounding boxes, BI-RADs scores, pathological diagnosis, and clinical information. The deeper annotations also allow for a wider variety of downstream applications such as lesion localization, semantic segmentation, explainable AI, and multimodal learning.

Despite these developments there are still many challenges to be addressed. The majority of publicly available datasets are from North America and Europe, and are limited in terms of demographic and ethnic diversity. There is also significant domain shift, due to the differences in the imaging equipment used, acquisition protocols and the standards used to annotate the images, which can limit the model performance when tested on other datasets outside the training set. Furthermore, the growing use of private institutional datasets contributes to the development of better models, but sacrifices reproducibility and standardized benchmarking between studies.

This review has pointed out that advanced learning algorithms are not only required but also the availability of large-scale, well-annotated and clinically representative breast cancer mammography datasets. Table 1 provides a comparison of the most common mammography datasets that are publicly available for use for AI applications for breast cancer detection. The transition from small datasets with film-based imaging (MIAS) to large datasets with FFDM imaging (VinDr-Mammo, OMI-DB) shows significant advances in data volume, annotation, and clinical data.

The primary issues are the absence of a standardized set of annotation protocols and the availability of detailed clinical metadata. While recent datasets have begun incorporating findings of lesion segmentation, BI-RADS assessments, and pathological findings, the variability in annotation standards makes it difficult to achieve reproducibility and to benchmark across datasets. Moreover, most data sets available are from Western populations, leading to demographic bias and lack of model generalization for populations not well represented by the available data. However, there are lack of repositories that offer the combination of large-scale data, comprehensive metadata, demographic diversity, and open access to the public.

Table 2 outlines the features that contribute to the success of a dataset in AI-related applications. Early datasets were mainly focused on achieving good classification performance using the image-level annotations. In contrast, current datasets provide richer annotations at the pixel level, BI-RADS assessment, and clinical information, enabling the application of more advanced methods, such as lesion detection and segmentation. Moreover, larger collections are better suited for pre-training large transformer-based networks, while smaller ones can be used for transfer learning and benchmarking purposes.

**Table 2:** Comparison of dataset characteristics relevant to AI development.

Characteristic	MIAS	DDSM	CBIS-DDSM	INbreast	VinDr	CMMD	OMI-DB	DMID
Large dataset	×	✓	✓	×	✓	✓	✓	×
Digital mammography	×	×	×	✓	✓	✓	✓	✓
Pixel-level annotation	×	×	✓	✓	×	×	✓	✓
Bounding boxes	×	✓	✓	✓	✓	×	✓	✓
BI-RADS labels	×	×	Partial	✓	✓	✓	✓	✓
Pathology labels	✓	✓	✓	✓	✓	✓	✓	✓
Clinical metadata	×	×	Partial	Partial	Partial	✓	✓	✓
Multi-center data	×	×	×	×	×	×	×	×
Suitable for CNNs*	Medium	High	High	High	Very high	High	Very high	Medium
Suitable for transformers*	Low	Medium	High	Medium	Very high	High	Very high	Low

^*^These ratings represent the authors' relative assessment of dataset suitability (refer section 2.4) and are not experimentally measured model-performance scores or ratings reported by the original dataset sources.

### 3.3 Key challenges in existing mammography datasets

Despite substantial progress in dataset development, several challenges continue to limit the clinical applicability of AI-based breast cancer detection systems. As summarized in Table 1, Table 3, publicly available datasets differ considerably in terms of size, annotation quality, imaging modality, and accessibility. While early datasets such as MIAS and INbreast provide valuable benchmark resources, their limited sample sizes restrict the training of modern deep learning and transformer-based architectures (refer to Figure 3). Conversely, larger repositories such as OMI-DB and VinDr-Mammo improve data diversity but present challenges related to heterogeneous imaging protocols and restricted accessibility.

**Table 3:** Strengths and limitations of major mammography datasets.

Dataset	Major strengths	Major limitations
MIAS	Simple benchmark dataset; widely adopted for algorithm evaluation; suitable for lightweight CNNs and traditional ML.	Very small dataset; limited demographic diversity; no lesion masks.
DDSM	Large benchmark dataset with pathology information; extensively used for CAD development.	Digitized film mammograms; inconsistent image quality; limited population diversity.
CBIS-DDSM	Standardized preprocessing; lesion segmentation; reproducible benchmarking.	Moderate dataset size; predominantly Western population.
INbreast	High-quality FFDM images; pixel-level annotations; good for segmentation research.	Limited number of patients; unsuitable for training data-hungry models.
VinDr-Mammo	Large modern FFDM dataset; BI-RADS assessment; lesion localization.	Single-country population; limited longitudinal information.
CMMD	Includes molecular subtype labels; supports precision medicine research.	No ROI annotations; limited institutional diversity.
OMI-DB	Very large dataset; longitudinal screening information.	Restricted access; heterogeneous imaging protocols.
DMID	Indian population; lesion annotations with clinical metadata; supports multiple AI tasks.	Limited public availability; still evolving.

**Figure 3:**
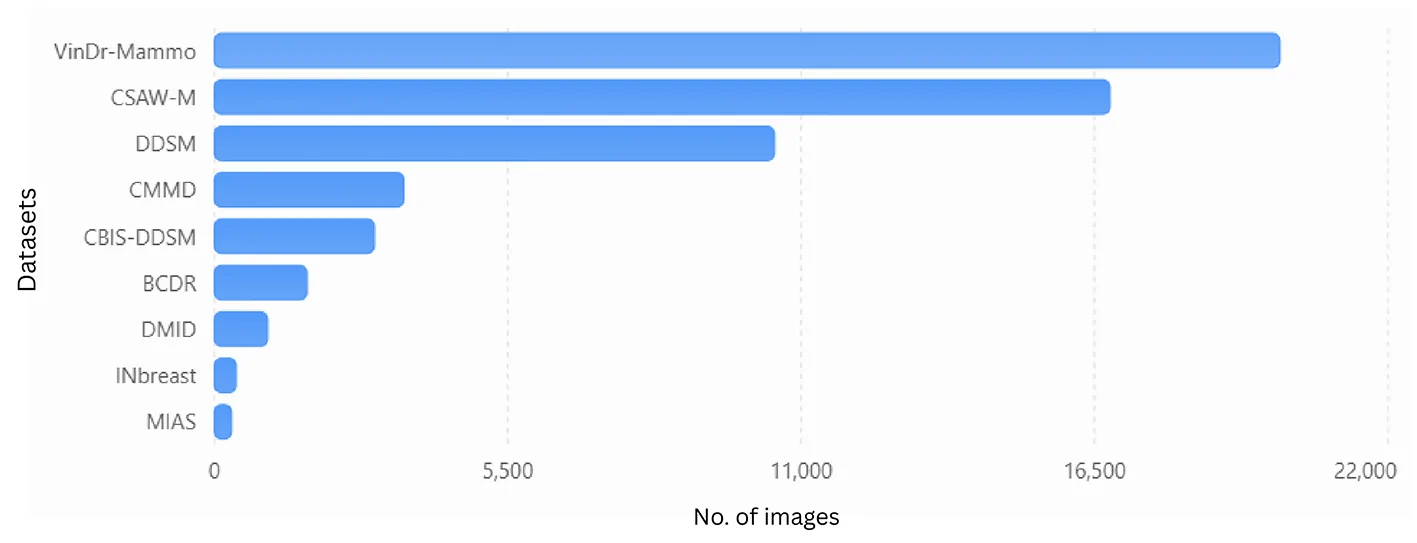
Comparison of major mammography datasets based on size.

The lack of standardized annotation protocols and the availability of comprehensive clinical metadata are the major limitations. Although recent datasets increasingly include lesion segmentation, BI-RADS assessments, and pathological findings, inconsistencies in annotation standards complicate reproducibility and cross-dataset benchmarking. Furthermore, most publicly available datasets are derived from Western populations, resulting in demographic bias and reduced model generalization when applied to under-represented populations.

## 4 Annotation standards and clinical labeling

The proper clinical annotation and standardization of breast cancer detection using mammograms is crucial for the development of computer-aided diagnosis (CAD) systems. This can have a major effect on the performance of traditional machine learning and deep learning techniques. If there are inconsistent or incorrect annotations, they can cause inaccurate learning, suboptimal generalization, and wrong clinical predictions. While there have been great advances in applying AI to mammography analysis, there is still a lack of standardized annotations.

### 4.1 BI-RADS standard

The American College of Radiology has created a standardized system called BI-RADS (Breast Imaging Reporting and Data System) for interpreting the results of a mammogram and categorizing mammograms by the probability that they will contain cancer. In addition to the categorical assessment, BI-RADS also includes a set of descriptors that characterize different aspects of the mammographic findings, such as lesion shape, margins, calcifications, and breast density, which can be used for the creation of standardized clinical labels for AI applications.

Several recent studies have incorporated BI-RADS annotations in creating the deep learning models for classification and risk prediction tasks (Gudhe et al., 2024; Rani et al., 2024). Standard BI-RADS annotations increase the consistency of annotations across different studies, allowing comparisons and benchmarking between various AI approaches.

### 4.2 Types of annotations in mammography datasets

Various annotation strategies are suitable for various AI tasks, and they directly affect the model complexity and diagnostic ability. Image-level labels are adequate for classification, while, for detection, the location of the lesions should be provided using bounding boxes, and, for segmentation, the precise pixel-level annotation should be provided. Thus, the granularity of the annotations is now a critical consideration when choosing AI architectures.

The image-level annotations are usually given for the whole mammogram and are used extensively for binary and multi-class classification models, such as in Arevalo et al. (2016) and Ayana et al. (2023). They are cheap to procure and require less space for classification but lack spatial data, which restricts their use for explainable AI and lesion localization.

Bounding box annotations are used to localize the lesions approximately and are the basis of object detection models like YOLO and Faster R-CNN (Hassan et al., 2024; Karaca Aydemir et al., 2025). They are superior to image-level labels under the following conditions: They are more interpretable, and they allow lesion detection but do not accurately segment lesion boundaries.

Pixel-wise annotations can precisely draw the outline of the lesion and play an important part in segmentation networks like U-Net and Mask R-CNN (El-Banby et al., 2024; Thakur et al., 2024). They are available with the highest level of annotation, with support for explanations, but cannot be acquired without significant expert effort, making them not scalable.

### 4.3 Impact of annotation quality on AI models

The quality of the annotations of the data has a strong influence on both the model selection, and the performance of diagnosis. The image level labels are mainly used for classification networks while the detection and segmentation architectures need more detailed annotations. Recent works show that multi-level annotated datasets can be used to train multi-task learning models that can perform multi-task classification, localization, and segmentation. Thus, more detailed annotation protocols provide greater model interpretability, enable explainable AI and enable clinical applicability. The relationship between annotation granularity and widely used AI architectures are summarized in Table 4.

**Table 4:** Mapping between annotation types and suitable deep learning models.

Annotation type	Description	Suitable models
Image-level Annotation	Global labels such as benign/malignant classification and BI-RADS categories	CNN, Vision Transformer (ViT), ResNet, and EfficientNet
Bounding Box Annotation	Localization of lesions using rectangular regions around suspicious areas	YOLO, Faster R-CNN
Pixel-level Annotation	Precise segmentation of lesion boundaries at the pixel-level	U-Net, U-Net++, and Mask R-CNN
Multi-level Annotation	Combination of image-level, region-level, and pixel-level annotations for comprehensive learning	Multi-task learning models, hybrid CNN–Transformer architectures

### 4.4 Clinical descriptors in mammography

Beyond image appearance, clinical parameters like breast density, lesion morphology, characteristics of the lesions in terms of calcification and architectural distortion can be helpful in providing additional information. These semantic descriptors are then gradually integrated with imaging features in current multimodal learning frameworks to further enhance diagnostic performance and interpretability (Jiang et al., 2022; Sahu et al., 2024; Gupta et al., 2025). But this type of data is not available in many public datasets, which restricts the creation of clinically aware AIs.

Recently, OncoVision, a multimodal AI for mammography that fuses mammographic images with clinical data with an “attention” framework has been developed. This approach segments the ROI based on the prediction of clinical descriptors like mass, calcification, breast density and BI-RADS categories. This framework also investigates the effect of late-fusion approaches, which involve dual-confidence scoring and attention-based visualizations, showing the potential of combining imaging and clinical information for a more comprehensive evaluation of the mammographic image (Ahmed et al., 2025).

### 4.5 Challenges in annotation and labeling

Despite considerable improvements, several challenges remain in mammography annotation. Inter-observer variability is one of the main challenge that continues to affect BI-RADS consistency, introducing label noise that reduces model robustness (Wang et al., 2020). Also, heterogeneous annotation formats across datasets hinder reproducibility and standardized benchmarking. Many publicly available datasets also lack comprehensive clinical metadata, limiting multimodal learning. Finally, obtaining high-quality pixel-level annotations remains labor-intensive and costly, motivating growing interest in weakly supervised, semi-supervised, and transfer learning approaches (Oza et al., 2024a; Khaled et al., 2025).

Therefore, quality annotation is important as dataset size to improve AI model performance. Inconsistencies in standard annotation across various datasets continue to affect models' generalization and benchmarking.

## 5 Machine learning and deep learning approaches

### 5.1 Traditional machine learning approaches

The traditional machine learning methods always relied on handcrafted texture, shape and statistical descriptors and classifiers like SVM, ELM. These methods proved that analysis of mammograms using computers was feasible and led to results comparable with those of other teams on relatively small benchmark datasets (Xie et al., 2016; Jadoon et al., 2017; Kaur et al., 2019). Their reliance on manual feature engineering, however, meant that they could not be scaled to be robust across datasets collected under different imaging protocols. The restrictions led to the shift toward end-to-end deep learning models that can learn discriminative representations directly from mammograms.

### 5.2 Deep learning approaches

The development of deep learning has drastically changed the analysis of medical images by enabling it to extract features automatically and learn hierarchical representations. One of the most recent and popular applications of deep learning models in the healthcare sector is the analysis of mammograms, particularly the use of Convolutional Neural Networks (CNNs).

#### 5.2.1 Convolutional Neural Networks (CNNs)

The CNNs eventually became the main architecture for mammography analysis due to their ability to learn the hierarchical image representations automatically. Further studies have helped to boost accuracy in deep networks, feature fusion, attention mechanisms, and ensemble learning, reaching an accuracy of more than 95% on benchmark datasets, as reported by (Arevalo et al., 2016; Li et al., 2019; Agnes et al., 2020; Jiang et al., 2022; Oza et al., 2023). However, they are still rather sensitive to the size and diversity of the data sets, and generalization across data sets is still a significant challenge.

#### 5.2.2 Transfer learning

Due to the lack of large comprehensive datasets with annotations, the use of transfer learning has become a popular strategy in breast cancer detection. Transfer learning helps to leverage pre-trained models trained on large-scale natural image datasets like ImageNet to improve feature representation, speed up convergence, and decrease the need for extensive labeled medical data for training.

The effectiveness of transfer learning for the classification task has been validated in several studies and has been shown to significantly boost the accuracy of classification across mammographic datasets, both in terms of feature extraction and avoiding overfitting (Huynh et al., 2016; Yala et al., 2019; Khamparia et al., 2021). This paradigm has been further extended recently by multi-stage transfer learning, adaptive fine tuning and Vision Transformer based transfer learning, resulting in greater robustness and cross-dataset generalization (Adedigba et al., 2022; Ayana et al., 2022, 2023). Despite such progress, transfer learning can still be affected by the domain mismatch, as models learned on natural images might not capture the nature of mammographic images.

#### 5.2.3 Object detection models

Besides the classification of a disease, accurate localization of a lesion is crucial for clinical decision support due to the visual observations of suspicious areas. Thus, the object detection models like YOLO and Faster R-CNN have gained popularity to perform automatic lesion detection in mammograms.

YOLO based methods have faster inference speed and are applicable to real-time screening, while Faster R-CNN usually achieves better localization accuracy, especially for small lesions, but at a higher computational cost (Al-Masni et al., 2018; Ribli et al., 2018). More recent works have integrated object detectors with transformer classifiers and attention mechanisms to boost lesion localization, diagnostic precision, and the interpretability of the models, thus strengthening their prospects for clinical use (Hassan et al., 2024; Karaca Aydemir et al., 2025).

#### 5.2.4 Segmentation-based approaches

Segmentation models are designed to distinguish the boundaries of a lesion at the pixel-level with detailed morphological information to aid diagnosis, treatment planning, and clinical interpretation. U-Net and its extensions continue to be the preferred architecture since they can effectively capture lesion boundaries while retaining fine spatial information.

The past few years have seen efforts to combine segmentation and detection/classification in a single CAD system for complete end-to-end analysis of the mammograms (Al-Antari et al., 2018). Incorporating attention mechanisms, feature fusion techniques, and multimodal learning has further enhanced the segmentation accuracy and robustness, though the availability of large annotated datasets remains constrained by the need for pixel-level annotations (Gupta et al., 2025).

#### 5.2.5 Hybrid and multi-stage models

Hybrid and multi-stage approaches combine complementary tasks (e.g., lesion detection, segmentation, and classification) into a single diagnostic pipeline. These methods attempt to increase diagnostic performance and better simulate the clinical process of radiologists by integrating multiple learning paradigms.

Recent research has also included ensemble learning, multimodal data fusion, transfer learning, and sequential decision-making for better robustness and generalization over various datasets (Al-Antari et al., 2018; Patil and Biradar, 2021; Pramanik et al., 2023; Atrey et al., 2024). Recent methods for semi-supervised learning fuse CNNs with recurrent networks or clustering methods or new emerging quantum inspired models to harness their complementary feature representations and attain competitive performance (Malebary and Hashmi, 2021; Azevedo et al., 2022). While these frameworks have always been reported to be highly accurate, the high complexity of their architectures and the high computational costs have been concerns to their routine clinical application.

#### 5.2.6 Emerging trends: transformer-based models

The transformer-based architectures offer a potential solution to CNNs, using self-attention to capture the long-range dependencies and global information in mammograms. In comparison to convolutional models, Vision Transformers (ViTs) can capture intricate spatial relationships and are therefore appealing for dense breast tissue analysis.

Recent experiments have shown that on benchmark mammography datasets, ViTs can achieve competitive classification performance and high AUC values, and hybrid CNN–Transformer architectures can further boost classification performance by leveraging local texture representation and global contextual learning of the images, as shown in Ayana et al. (2023), Umamaheswari and Babu (2024), and Hassan et al. (2024). But the transformer models generally require much more data to train than CNNs, which makes them impractical in clinical settings where access to data is limited. Hence, the current research is increasingly centered on designing efficient architectures for transformers that achieve accuracy, cost, and generalization.

### 5.3 Performance metrics

Machine learning and deep learning models are usually assessed by standard quantitative metrics. They are accuracy, sensitivity (recall), specificity, F1-score, and the Area Under the receiver operating characteristic Curve (AUC).

The measures of accuracy, sensitivity, and specificity are used to determine the overall correctness, correctness in identifying positive and negative cases, respectively. F1-score offers a compromise between precision and recall, especially when there is an imbalance of the dataset. AUC is largely considered a strong predictor of model performance at various decision levels.

Other researchers, like Ribli et al. (2018) reported high AUC values, indicating that deep learning-based CAD systems are effective. Recent studies by Oza et al. (2023), Gupta et al. (2025) also demonstrate that there are steady changes in performance indicators as complex architectures are implemented.

### 5.4 Key observations

According to the literature reviewed, it is possible to outline several significant tendencies of the breast cancer detection system development. The models that dominate this field of research are CNN-based models, which can learn hierarchical and discriminative features of medical images. Moreover, transfer learning has become an essential method to deal with small size of annotated datasets, allowing models to utilize the experience of large-scale pre-trained networks.

Moreover, detection and segmentation-based methods have become of great interest because they enhance interpretability and offer clinically relevant localization of lesions. Multi-stage models and hybrid models have been explored more widely, providing a finer-grained solution to the problem of diagnostics by incorporating classification, detection, and segmentation. Also, model performance is highly dependent on the data quality, quantity, and diversity used in training and testing the models. In addition, there is great potential for further research using transformer architectures, especially in capturing long-range dependencies.

### 5.5 Evolution of automated breast cancer detection

Breast cancer detection has come a long way from feature-based handcrafted machine learning approaches to deep learning and even more recently to transformer-based approaches. Most early works were based on manually designed texture and morphological descriptors and classic classifiers like SVMs and ELMs, which showed the potential of computer-aided diagnosis but were limited in feature representation and cross-dataset generalization (Xie et al., 2016; Jadoon et al., 2017). The paradigm was changed with the introduction of CNNs, which allowed a more end-to-end learning of features, and transfer learning, which enhanced the performance of data-constrained medical imaging applications such as Arevalo et al. (2016), Huynh et al. (2016).

The focus of recent research has shifted to hybrid CNN–Transformer models, ensemble learning, multi-stage diagnostic frameworks, and privacy-preserving methods like federated learning (Oza et al., 2023; Ayana et al., 2023; Dharani Devi et al., 2024; Khaled et al., 2025). Many studies describe an accuracy above 98% on benchmark datasets; however, the accuracy falls below this threshold on the external clinical datasets, as different acquisition protocols, patient demographics and annotation quality exist (Liu et al., 2024). Consequently, current research increasingly emphasizes robust generalization, explainability, and clinically validated AI systems rather than incremental improvements in benchmark accuracy.

Table 5 and Figure 4 identifies the change in the manner of mammography-based breast cancer detection methods, as it is evident that the traditional approaches of machine learning were replaced by deep learning models, with the recent tendencies toward the adoption of hybrid and transformer-based architectures.

**Table 5:** Representative AI-based approaches for mammography-based breast cancer detection.

References	Dataset	AI technique	Task	Key contribution	Limitation
Traditional machine learning					
Xie et al. (2016)	DDSM	Feature Selection + ELM	Classification	Established a baseline ML framework using handcrafted features.	Requires manual feature engineering.
Jadoon et al. (2017)	MIAS	Gabor Features + SVM	Classification	Demonstrated effective texture-based mammogram classification.	Limited generalization across datasets.
Kaur et al. (2019)	MIAS	Hybrid ML	Classification	Improved feature representation through feature fusion.	Performance depends on handcrafted descriptors.
Deep learning (CNN-based models)					
Carneiro et al. (2017)	INbreast, DDSM	CNN (AlexNet Variant)	Classification	Introduced end-to-end CNN learning for mammography.	Requires lesion annotations.
Li et al. (2019)	DDSM	Deep CNN	Classification	Demonstrated robust automatic feature learning.	Sensitive to dataset size and diversity.
Cao et al. (2020)	DDSM	Optimized CNN	Classification	Improved classification using deeper CNN architectures.	High computational cost.
Mokni and Haoues (2022)	DDSM, INbreast	CADNet157 (ResNet152)	Classification	Enhanced performance through fine-tuned deep CNNs.	Requires large annotated datasets.
Transfer learning					
Huynh et al. (2016)	Private	Transfer Learning	Classification	Demonstrated effective learning with limited training data.	Domain mismatch from natural images.
Ruchai et al. (2021)	MIAS	Transfer Learning	Classification	Showed effectiveness of pretrained CNNs with augmentation.	Performance depends on preprocessing quality.
Khamparia et al. (2021)	CBIS-DDSM	Transfer Learning + CNN	Classification	Improved accuracy and computational efficiency.	Requires careful model adaptation.
Ayana et al. (2022)	DDSM, MIAS, INbreast	Multi-stage Transfer Learning	Classification	Reduced dependence on patch extraction.	Complex training pipeline.
Detection and segmentation					
Al-Masni et al. (2018)	DDSM	YOLO + CNN	Detection	Enabled real-time lesion detection and classification.	Reduced sensitivity for small lesions.
Ribli et al. (2018)	INbreast	Faster R-CNN	Detection	Accurate localization of suspicious regions.	High computational complexity.
Sarangi et al. (2021)	MIAS	Threshold-based Neural Network	Segmentation	Improved lesion boundary delineation.	Requires accurate preprocessing.
Jiang et al. (2022)	CBIS-DDSM, INbreast	EfficientNet + PAA	Multi-stage CAD	Integrated detection and classification.	Complex architecture.
Hybrid and emerging models					
Chakravarthy and Rajaguru (2022)	DDSM, MIAS, INbreast	ResNet18 + ELM	Classification	Combined deep learning with optimization techniques.	High training complexity.
Ayana et al. (2023)	DDSM	Vision Transformer	Classification	Demonstrated global-context learning using self-attention.	Requires large-scale training data.
Tan et al. (2023)	CBIS-DDSM	Federated Learning	Classification	Improved privacy-preserving distributed learning.	Communication overhead.
Sahu et al. (2024)	mini-DDSM	Ensemble CNN	Classification	Improved robustness through feature fusion.	Higher inference cost.
Kalpana and Selvy (2024)	INbreast	Ensemble Learning	Classification	Enhanced feature selection and classification accuracy.	Model complexity.
Thakur et al. (2024)	DDSM	Reinforcement Learning + CNN	Classification	Integrated reinforcement learning for adaptive diagnosis.	Longer training time.
Khaled et al. (2025)	INbreast	Ensemble Transfer Learning	Classification	Improved generalization through ensemble transfer learning.	Computationally expensive.

**Figure 4:**
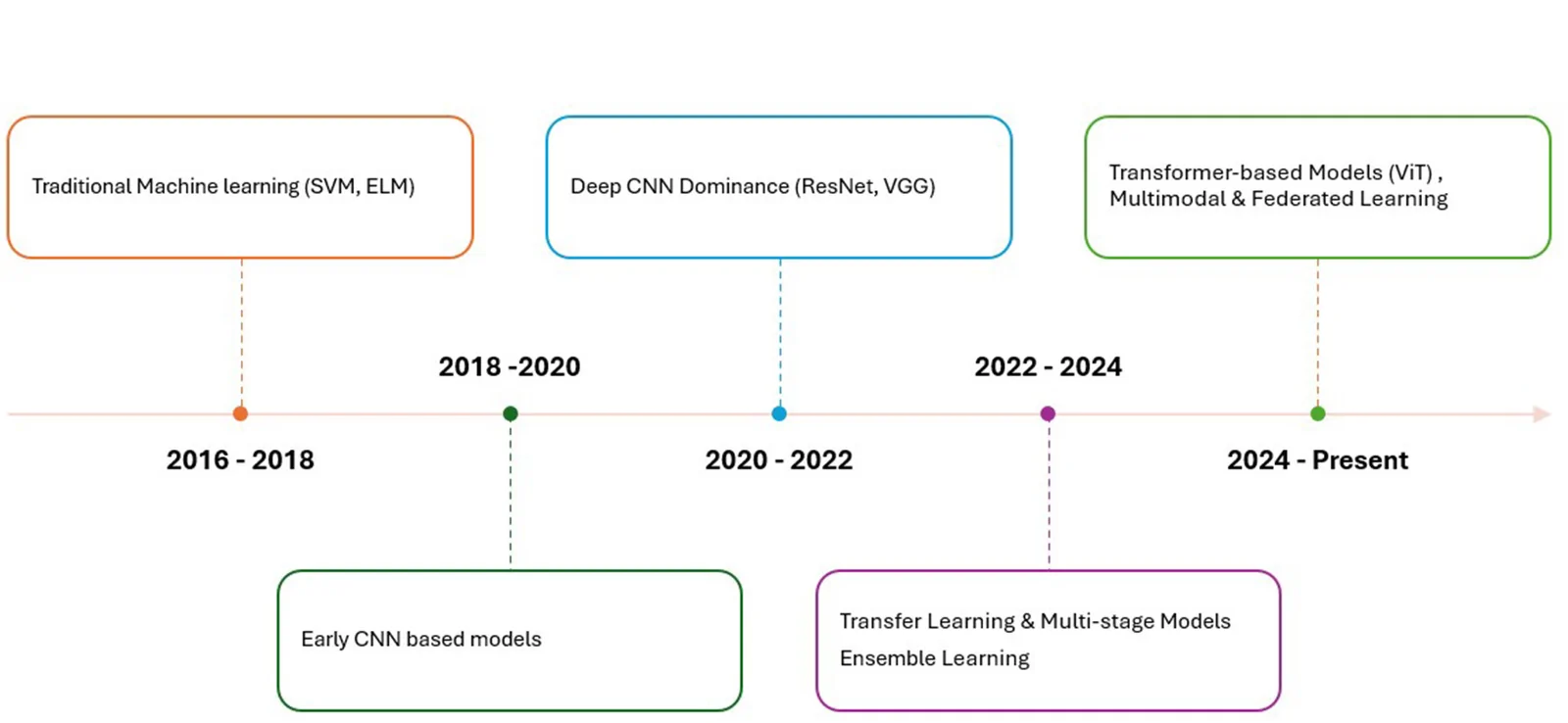
Evolution of breast cancer detection techniques.

### 5.6 Comparative analysis of state-of-the-art models

In order to offer a more in-depth insight into the effectiveness of models in various tasks, the section will provide a comparative analysis of the state-of-the-art deep learning models applied to mammography-based breast cancer detection in classification, detection, and segmentation tasks.

#### 5.6.1 Classification models

CNN-based architectures continue to dominate mammogram classification owing to their strong feature extraction capability and the availability of pretrained weights. Vision Transformers further improve global context modeling but generally require substantially larger training datasets. Recent hybrid CNN–Transformer models attempt to combine local texture representation with long-range contextual information, leading to improved robustness and cross-dataset performance.

#### 5.6.2 Detection models

Object detection models aim to localize suspicious lesions by maintaining diagnostic accuracy. Faster R-CNN has higher localization accuracy, but it is not computationally efficient, while the YOLO-based detectors have high computational speed, which is beneficial for real-time screening, but the accuracy may be lower due to the detection of small lesions. Detection and classification are increasingly treated as a single problem in recent studies, in order to achieve greater clinical usability.

#### 5.6.3 Segmentation models

To improve interpretability for clinical assessment, the segmentation models, for example, U-Net variants, are used to obtain more accurate detection of lesion boundaries. Although these models give better morphological analysis, their dependence on pixel-level annotations limits the availability of large training datasets. This is not the case for attention-based and multi-task architectures, which can achieve higher segmentation accuracy. Table 6 provides an overview of the features, strengths, weaknesses, and growth of some of the most popular models for the various tasks.

**Table 6:** Comparative analysis of deep learning models for mammography-based breast cancer detection.

Task	Model	Advantages	Limitations	Performance
Classification	CNN (ResNet, DenseNet, and EfficientNet)	Strong feature extraction; transfer learning; widely validated	Limited global context modeling	Accuracy > 90%
	Vision Transformer (ViT)	Captures global dependencies; strong contextual learning	Data-hungry; computationally expensive	AUC > 0.95
	Hybrid CNN–Transformer	Combines local + global features; improved generalization	Complex design; high training cost	Strong cross-dataset performance
Detection	YOLO (v5–v8)	Real-time; fast inference; deployment-friendly	Lower sensitivity for small lesions	High precision; moderate recall
	Faster R-CNN	High accuracy; strong small-object detection	Slow inference; high compute cost	High recall + precision
	RetinaNet	Handles class imbalance (Focal Loss)	Slower than YOLO; tuning complexity	Balanced precision–recall
Segmentation	U-Net/U-Net++	Precise boundaries; high interpretability	Requires pixel-level annotations	Dice > 0.85
	Mask R-CNN	Detection + segmentation in one framework	Computationally expensive	High multi-task accuracy
	Attention U-Net	Focuses on relevant regions; improved segmentation	Increased model complexity	Improved dice and IoU

## 6 Geographic distribution and bias analysis

Variety and representativeness of datasets used in training are two key elements of the performance and reliability of AI models, particularly in medical imaging. Geographic distribution is a very crucial factor in the generalization and clinical use of machine learning models in breast cancer detection through mammography. Even though the development of artificial intelligence in healthcare has been growing quickly, current literature indicates a strong disparity between the representation of datasets in various areas. Figure 5 shows the large-scale and public datasets available around the world.

**Figure 5:**
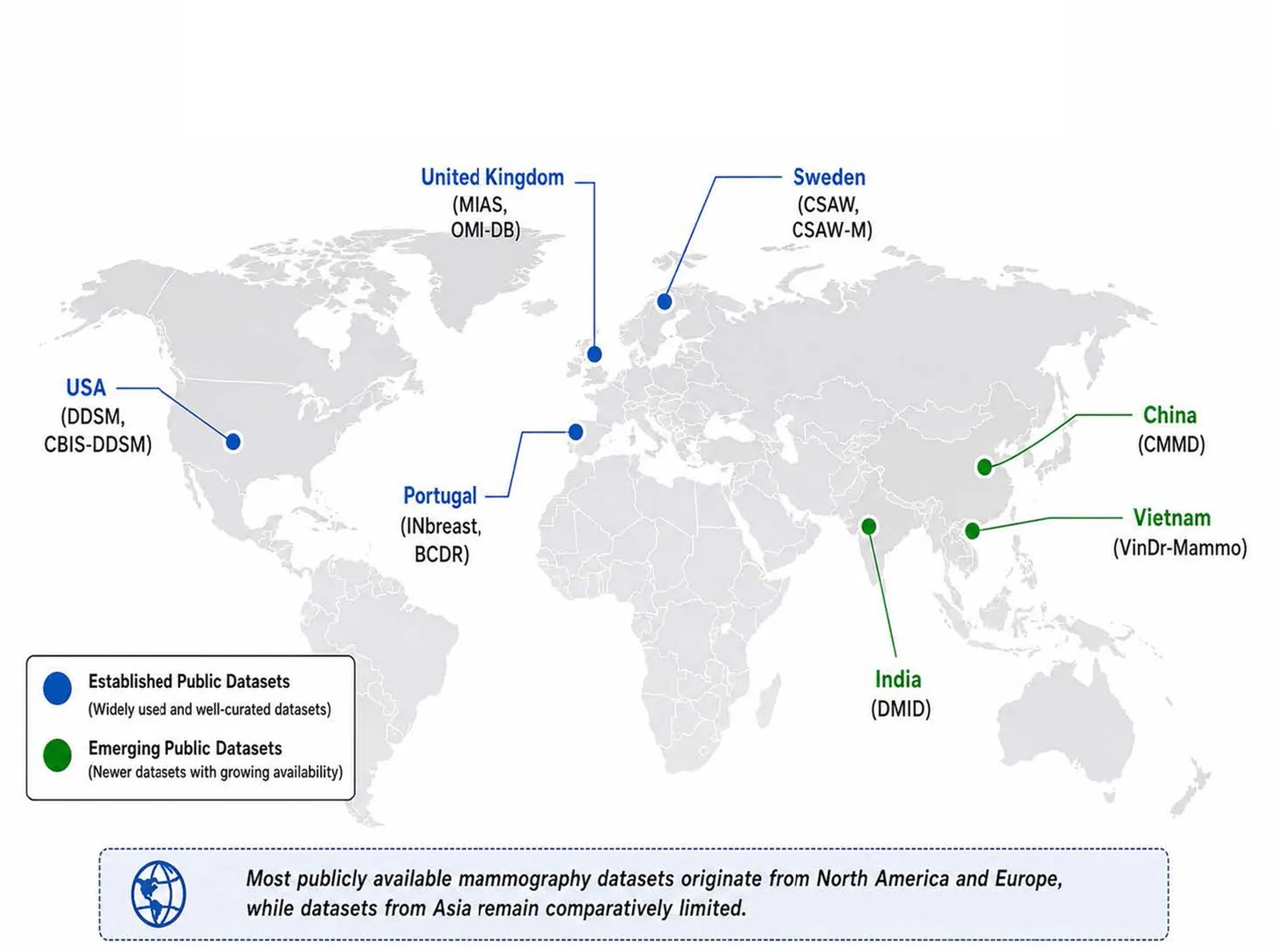
Global distribution and availability of mammography datasets.

### 6.1 Global distribution of mammography datasets

Review of literature revealed that there was a high geographic bias in the mammography datasets as most of the studies used North American and European data sets, primarily DDSM/CBIS-DDSM, MIAS, and INbreast. Other datasets like VinDr-Mammo, CMMD, and DMID, on the other hand, are less commonly used. The change in the amount of datasets used, from 2016 to 2025, is illustrated in Figure 2, with a steady increase in newer datasets and repositories of private institutional datasets, although the number of established datasets like MIAS and DDSM is still substantial.

These institutional datasets can provide clinically relevant data but their restricted accessibility limits reproducibility and standardized benchmarking (Huynh et al., 2016; Mohamed et al., 2018; Atrey et al., 2024; Gupta et al., 2025). Overall, the literature remains dependent on a relatively limited number of geographically concentrated datasets.

### 6.2 Geographic underrepresentation in mammography datasets

The literature study indicates that certain populations are geographically underrepresented, especially those from Africa, South America, and parts of Asia, who lack mammography datasets available in public is significant since population characteristics, breast density, disease prevalence, screening methods, imaging modality, and acquisition protocols may vary (Atrey et al., 2023; Hassan et al., 2024).

One example of such underrepresentation is India which, despite the heavy burden of breast cancer, still lacks datasets with appropriate representation of demographic and imaging diversity. DMID represents an important emerging database with lesion annotation and clinical metadata, however, its geographical and institutional diversity are not sufficiently covered.

### 6.3 Impact of geographic bias on model performance

Geographic and demographic variations may lead to the domain shift that will decrease the ability of a model to generalize. Variations in breast density, imaging properties, preprocessing, and disease distribution might affect the appearance and detection of abnormalities on mammograms. It has been demonstrated by the literature that models are sensitive to variations in the datasets and may demonstrate decreased performance when applied to independent datasets (Shen et al., 2019; Ribli et al., 2018; Oza et al., 2023; Karaca Aydemir et al., 2025).

Thus, models are able to perform well in one dataset does not imply their generalization capability. External validation on geographically diverse datasets is required.

### 6.4 Consequences for clinical deployment

The geographic bias can affect the reliability of AI systems when deployed in diverse populations that differ from their training data, potentially increasing false-positive or false-negative predictions. This can decrease the confidence of healthcare providers, particularly those from underrepresented groups.

Thus, models designed for clinical application should be validated in an external setting in a variety of populations and healthcare settings. The evaluation is crucial to verify the feasibility of the reported performance in the real clinical context (Hassan et al., 2024; Gupta et al., 2025).

## 7 Open challenges and research gaps

The machine learning and deep learning methods are a great advancement in the detection of breast cancer. Key challenges that impact the efficiency and usability of AI-driven health care systems include the construction of datasets, training machine learning models, clinical validation, and implementation. In this section, an overview of the primary problems emerged in this study is given. Table 7 presents the list of gaps found in literature.

**Table 7:** Summary of key research gaps and future directions.

Research Gap	Evidence from the Literature	Future Direction
Small and homogeneous datasets	Limited size of benchmark datasets such as MIAS and INbreast restricts model generalization.	Develop large-scale, multi-center, and demographically diverse datasets.
Annotation inconsistency	Heterogeneous annotation formats and inconsistent adoption of BI-RADS across datasets hinder reproducibility.	Establish standardized annotation protocols with unified BI-RADS labeling.
Cross-dataset generalization	Significant performance degradation is observed when models are evaluated on external datasets.	Promote external validation, domain adaptation, and cross-dataset benchmarking.
Limited clinical translation	Most studies rely on retrospective benchmark datasets with limited prospective clinical validation.	Conduct multi-center clinical trials and integrate AI into routine clinical workflows.
Privacy and data sharing	Dependence on institutional datasets limits reproducibility and collaborative model development.	Adopt federated learning and privacy-preserving AI frameworks.

### 7.1 Dataset and annotation challenges

According to analysis conducted in Sections 3 and 4, the major constraint of the use of AI technologies in the diagnosis of breast cancer is the quality of the datasets. Although some open datasets like MIAS, CBIS-DDSM, and INbreast have been open to researchers and provided some useful methodologies to aid in the development of methods, these datasets still lacked sufficient patient numbers, a lack of demographic diversity, and inconsistent annotations that may overfit the model (Wang et al., 2020).

The standardization of annotation methods is another problem that should be solved. The improvement of datasets through adding more details like BI-RADS annotations, lesion segmentation, etc. did not solve this problem. In addition, the scarcity of multimodal data that combines imaging with clinical information remains a significant hurdle in the advancement of clinically informed systems in AI for breast cancer diagnosis and treatment (Atrey et al., 2023; Gupta et al., 2025).

### 7.2 Model generalization challenges

Although there has been great progress in classification accuracy, model robustness is a great concern. Due to variations in the imaging protocol, the annotation process, patient demographics, etc., many deep learning models perform well on a particular benchmark dataset, but the performance drops significantly when applied to independent datasets (Shen et al., 2019; Wang et al., 2020). While transfer learning, ensemble learning and domain adaptation partially solve this problem, they do not solve the problem of dataset bias completely.

Besides, the class imbalance and lack of model interpretability still hinder the clinical application. Recent advances such as explainable AI, uncertainty estimation and multi-task learning have shown potential but are not yet widely adopted in clinical practice in mammography.

### 7.3 Clinical translation challenges

Although there has been great progress in classification accuracy, model robustness is a great concern. Due to variations in the imaging protocol, the annotation process, patient demographics, etc., many deep learning models perform well on a particular benchmark dataset but the performance drops significantly when applied to independent datasets (Shen et al., 2019; Wang et al., 2020). While transfer learning, ensemble learning and domain adaptation partially solve this problem, they do not solve the problem of dataset bias completely.

Besides, the class imbalance and lack of model interpretability still hinder the clinical application. Recent advances such as explainable AI, uncertainty estimation and multi-task learning have shown potential but are not yet widely adopted in clinical practice in mammography.

### 7.4 Future research priorities

Based on the results of this review, the future progression of AI in mammography will require a change toward more scalable and standardized datasets and standardized external validation over increasingly complex model architectures. Geographical diversity of benchmark sets should thus be promoted, standardized protocols for annotations should be developed, multimodal clinical data should be included, and common evaluation frameworks should be set up. Solving these challenges will enhance model robustness, reproducibility, and clinical applicability and facilitate equitable deployment in various healthcare settings.

## 8 Need for a geographically diverse benchmark dataset

This review suggests that there is a need for benchmark datasets that more accurately reflect geographic, demographic and clinical diversity to enable the development of reliable AI systems for mammography. Presently, most of the literature is focused on specific geographic areas, and fewer data sets are available for Africa, South America and part of Asia. This imbalance makes it difficult to evaluate the generalizability of the model to other populations and healthcare settings.

### 8.1 Evidence from existing literature

According to the analyzed studies, datasets majorly contribute to the effectiveness of the AI systems. While CNN, transfer learning, ensemble, and Vision Transformer-based models usually perform well on benchmark datasets, it is not clear whether they will do the same on other datasets (Shen et al., 2019; Wang et al., 2020; Oza et al., 2023). Differences in performance are likely due to different populations, imaging protocol, equipment, and disease prevalence.

In addition to improvements in model architecture, the improvement of diversity and representation in benchmark datasets becomes an important aspect due to the above findings. A good benchmark dataset, therefore, should allow assessing several populations.

### 8.2 Requirements for a comprehensive benchmark dataset

Based on the reviewed literature, future benchmark datasets should incorporate several key characteristics:

**Multi-center and geographic diversity:** Data should be gathered from a number of institutions and geographic locations to reflect population, clinical practice and imaging system variations.

**Standardized annotation:** To facilitate meaningful comparison and increase reproducibility, consistent diagnostic labels, BI-RADS assessments, localization of the lesions, and, if available, segmentation annotations should be provided.

**Clinical metadata:** Information such as age, breast density, pathology findings, and follow-up outcomes can support the development and evaluation of clinically relevant and multimodal AI systems.

**Privacy-preserving collaboration:** Federated and other privacy-preserving approaches can facilitate collaboration between institutions while reducing the need to centralize sensitive patient data.

**Standardized benchmarking:** Clearly defined data splits, evaluation protocols, and common performance metrics are essential for fair comparison of AI models.

### 8.3 Relevance to India and other underrepresented regions

In the wider picture, India is one of several countries where there is scope to have more representative data on mammography to supplement global benchmarking. Likewise, better representation of the under-represented groups of Africans and South Americans would enhance the geographic and demographic representation of future resources.

Therefore, the development of regionally representative datasets should be considered as part of the national effort to build a national dataset, rather than as a requirement for India. Resources like this would help accurately evaluate cross-population generalization, help develop AI in an equitable way, and offer more evidence to the clinical viability of the use of AI systems in mammography.

### 8.4 Expected impact

A cross-disciplinary, standardized benchmark would provide meaningful comparisons of the performance of AI models, promote external validation, and enhance the understanding of performance differences between individuals and populations, and other health care contexts. The ability to represent data from beyond just the dominant regions would enable their integration into more effective, widely applicable and fair breast cancer detection systems using AI.

## 9 Conclusion

This review provided a comprehensive survey of breast cancer detection using mammography, covering the interaction between publicly available datasets, annotation approaches, and AI models. This work focused on the various factors of the dataset that profoundly affect the robustness of the models, their generalization, and their clinical applicability, such as size, annotation quality, demographic diversity, and clinical metadata. This comparison shows that, apart from the ever more complex architectures, it is also the quality and representativeness of the data used to train the AI that are contributing to its improvement.

The review also gathered and consolidated the latest advancements in classical machine learning, deep learning, transfer learning, transformer networks, multimodal learning, explainable AI, and privacy-preserving techniques and summarized their strengths, as well as identifying their current challenges. Despite the excellent diagnostic accuracy often seen in the literature on benchmark datasets, there is growing evidence that cross-dataset generalization, external validation, annotation variability, and demographic diversity are still significant challenges for reliable clinical use. The results indicate that the emphasis on future research should be on standard benchmarking, multi-center validation, and clinically relevant datasets, in addition to architectural enhancements.

One of the key insights of this review is the discovery that the current obstacle for the clinical deployment of breast cancer detection AI systems is the existence of a large number of challenges related to the datasets. The evidence presented in this paper suggests that there is a need for the development of a broad, well-annotated clinical benchmark dataset with comprehensive clinical metadata and uniformly applied annotation protocols on a large scale, with a diverse demographic profile. These resources would aid in fair comparison of models, enhance model reproducibility, and help to evaluate the models in various healthcare settings.

Lastly, it is noted that a more geographically representative benchmark mammography database is needed to overcome the current lack of global populations in public repositories. The data has the potential to facilitate research in the country and the development of multiple breast cancer detection tools based on artificial intelligence that are more reliable, balanced, and clinically viable, whether they are based on conventional or digital mammograms. These data can be leveraged to develop a more accurate, fair and clinically relevant breast cancer detection system, regardless of the type, and for the development of different AI-based tools for breast cancer detection. Based on the high-quality datasets and the new AI paradigms, future studies could build on the multimodal learning, explainable AI and federated learning, to move toward trusted, widely deployed computer-aided diagnosis systems.
